# Integrative bioinformatics and experimental analysis revealed TEAD as novel prognostic target for hepatocellular carcinoma and its roles in ferroptosis regulation

**DOI:** 10.18632/aging.203853

**Published:** 2022-01-25

**Authors:** Xinxin Ren, Xiang Wang, Yuanliang Yan, Xi Chen, Yuan Cai, Qiuju Liang, Bi Peng, Zhijie Xu, Qingchun He, Fanhua Kang, Jianbo Li, Wenqin Zhang, Qianhui Hong, Jinwu Peng, Muzhang Xiao

**Affiliations:** 1 Center for Molecular Medicine, Xiangya Hospital, Key Laboratory of Molecular Radiation Oncology of Hunan Province, Central South University, Changsha 410008, Hunan, China; 2 Department of Pharmacy, Xiangya Hospital, Central South University, Changsha 410008, Hunan, China; 3 Department of Pathology, Xiangya Hospital, Central South University, Changsha 410008, Hunan, China; 4 National Clinical Research Center for Geriatric Disorders, Xiangya Hospital, Central South University, Changsha 410008, Hunan, China; 5 Department of Emergency, Xiangya Hospital, Central South University, Changsha 410008, Hunan, China; 6 Department of Emergency, Xiangya Changde Hospital, Changde 415000, Hunan, China; 7 Department of Pathology, Xiangya Changde Hospital, Changde 415000, Hunan, China; 8 Department of Burn and Plastic Surgery, Xiangya Hospital, Central South University, Changsha 410008, Hunan, China

**Keywords:** TEAD family, hippo pathway, hepatocellular carcinoma, ferroptosis, immune infiltration

## Abstract

Objective: Transcriptional enhanced associate domain (TEAD) family consists of four members TEAD1/2/3/4 that regulate cell growth, stem cell functions and organ development. As the downstream of Hippo signaling pathway, TEAD family is involved in the progression of several cancers. However, the precise biology functions of TEAD family in hepatocellular carcinoma (HCC) have not been reported yet.

Methods: We apply bioinformatics analysis based on databases including UALCAN, Oncomine, GEPIA, Kaplan-Meier plotter, WebGestalt, cBioPortal, TIMER2.0, and *in vitro* experimental evidence to identify the exact roles of TEAD family in HCC.

Results: The results indicated that TEAD2/4 were significantly upregulated in HCC compared with normal tissues. Downregulated of TEAD2 could promote the death of HCC cells through inducing ferroptosis by iron accumulation and subsequent oxidative damage. According to the Kaplan-Meier plotter database, we found that the high expression of TEAD2 was significantly associated with poor disease-specific survival, overall survival, progression-free survival and relapse-free survival. In aspect of cancer immunity, Tumor Immune Estimation Resource algorithm showed that the expression of TEAD family members was obviously related to multiple of infiltrating immune cells including macrophages, neutrophils, dendritic cells, B cells, CD8+ T cells and CD4+ T cells. Finally, we conducted the functional enrichment analysis including protein-protein interaction network, gene ontology enrichment analysis and Kyoto Encyclopedia of Genes and Genomes pathway based on the TEAD family-associated coexpression genes.

Conclusion: The study provided deep insight information of TEAD family in the diagnostic and prognostic evaluation of HCC patients.

## INTRODUCTION

The occurrence of liver cancer is rising gradually [1]. Research estimates that there will be over 1 million individuals that develop liver cancer per year by 2025 [2]. Hepatocellular carcinoma (HCC) occupies about 90% of liver cancer patients [3]. At present, we apply chemoradiotherapy and surgery to deal with the early stage of HCC in clinical and obviously improve the patients’ condition [4]. However, the majority of patients are diagnosed in advanced stages [5]. In addition, the molecular pathogenesis of HCC varies on the basis of the different etiologies and genotoxic insults [6]. Then, searching for novel molecular targets are crucial for the diagnosis and prognosis of HCC.

Transcriptional enhanced associate domain (TEAD) family take vital effects in the organism [7]. The TEAD family contains four members (TEAD1, TEAD2, TEAD3 and TEAD4), which each have tissue-specific roles, including trophectoderm, cardiogenesis, lineage determination and neural development [8]. TEAD family is regarded to be primarily combine to the DNA and it is also the downstream nuclear effectors of the Hippo pathway [9]. As we all know, Hippo pathway plays essential roles in cell proliferation through its downstream target genes, which has a crucial impact on tumorigenesis [10, 11]. Therefore, as reported, TEAD family was strongly related to the development of cancer and had an influence on several cancers including prostate cancer, ovarian cancer and lung cancer [12–14]. Moreover, YAP/TAZ could promote HCC cells to overcome sorafenib-induced ferroptosis in a TEAD-dependent manner, which suggested that there was a relationship between TEAD family and HCC [15]. Therefore, the roles of TEAD family in HCC are urgent to be illustrated.

Ferroptosis is always triggered by lipid oxidation [16]. Ferroptosis is involved in the development of several tumors, including HCC [17, 18]. Hippo signaling pathway is mediated by YAP) and TAZ, which interacted with TEAD proteins for gene expression regulation [19]. At present, YAP/TAZ has been found to be as the novel determinants of ferroptosis. In addition, YAP promotes ferroptosis via the E3 Ligase SKP2 in renal and ovarian cancer cells [20, 21]. However, the relationship between TEAD and ferroptosis has not been illustrated.

In the study, using bioinformatics databases and experimental verification, we discussed the effects of TEAD family in HCC (Supplementary Table 1). Furthermore, the potential as diagnostic biomarkers and prognostic targets were evaluated. Moreover, downregulated the expression of TEAD2 could promote ferroptosis in HCC cell lines. Through the comprehensive analysis, TEAD family members, especially TEAD2, contributed to helping clinicians proved better medical services for HCC patients.

## MATERIALS AND METHODS

### Cell culture

The normal hepatocyte HHL-5 and HCC parental cell lines PVTT, QGY, Huh7, MHCC97H, 7404 and 7721 were kindly provided by Professor Yuezhen Deng (Central South University, China). QGY cells were cultured by using 1640 medium (Gibco, Invitrogen), while other cells were cultured in DMEM, which contained 10% fetal bovine serum (FBS, Gibco) and 1% penicillin and streptomycin, incubation under the condition at 37°C with 5% CO_2_.

### Antibodies and chemicals

Antibodies and chemicals were employed in the study: TEAD2 (21159-1-AP, Proteintech), Actin (66009-1-Ig, Proteintech), erastin (B1524, APExBIO), RSL3 (B6095, APExBIO), Ferrostatin-1 (A4371, APExBIO), Z-VAD-FMK (A1902, APExBIO), Necrostatin-1 (A4213, APExBIO).

### Transfections

Lipofectamine 3000 was employed to perform siRNA transfection based on the manufacture’s protocol. The sense sequences of siTEAD2 were settled bellow: siTEAD2-1-GAGTGAGCAGCCAGTATGA, siTEAD2-2-GGTTGCAGCTGGTAGAGTT.

### qPCR

The PrimeScriptTM RT reagent kit (6210, Takara) was applied to converted the total RNA to cDNA. The qPCR assay was conducted with SYBR green kit (1725121, Bio-Rad), and 18S rRNA was employed for an internal control. We listed the primer sequences of TEAD family in Table 1 and confirmed the relative expression levels.

**Table 1:** The primer sequences of *TEAD* family involved in RT-PCR.

**Primers**	**Forward sequences**	**Reverse sequences**
TEAD1	ATGGAAAGGATGAGTGACTCTGC	TCCCACATGGTGGATAGATAGC
TEAD2	CTTCGTGGAACCGCCAGAT	GGAGGCCACCCTTTTTCTCA
TEAD3	TCATCCTGTCAGACGAGGG	TCTTCCGAGCTAGAACCTGTATG
TEAD4	GAACGGGGACCCTCCAATG	GCGAGCATACTCTGTCTCAAC

### Gene expression profiling interactive analysis (GEPIA)

GEPIA is a database used to provide numerous GTEx and TCGA data [22]. We explored the level of TEAD family member in HCC tissues compared with normal tissues.

### UALCAN

UALCAN is a public web tool employed for exploring gene expression and survival information [23]. We evaluated TEAD family expression in HCC patients.

### GE-mini

GE-mini database is generally used for the basic simple analysis of gene mutations, co-expressed/co-mutated genes [24]. We discussed the level of TEAD in HCC.

### Oncomine 3.0

Oncomine database integrates lots of data for the bioinformatic analysis [25]. From the database, the level of TEAD family in HCC patients was tested.

### Western blot

The protein concentration was tested and the separation operation is then performed. The membranes were incubated with the corresponding primary antibodies at 4°C overnight. Next day, the secondary antibodies were kept together with the membranes for 1–2 h and chemiluminescence reagent was applied to detect the immunoreaction signals.

### Cell viability assay

Cells were cultured with a density of 900 cells/well in a 96-well plate (). Then, the 10% CCK8 was added to the medium. The absorbance was calculated at 450 nm.

### Reactive oxygen species (ROS) assay

ROS levels were tested applying DCFDA/H2DCFDA Kit (ab113851, Abcam). We used the DCFDA Solution to stain cells away from light. Next, we employed the microscopy to detect fluorescence intensity.

### Iron assay

We used the iron assay kit (ab83366, Abcam) to test the Fe^2+^ level. Generally, 1 × 10^6^ cells were mixed together with 100 μl iron assay buffer. Then, the iron reducer was added and incubated for 30 min. Subsequently, the iron probe was added, then, tested at 593 nm.

### Malondialdehyde (MDA) assay

A Lipid Peroxidation Kit (MAK085, Sigma) was used to quantify the MDA concentration. The thiobarbituric acid (TBA) was applied to test the amounts of MDA at 531 nm.

### Kaplan-Meier plotter

Kaplan-Meier plotter is conducted based on gene chip and RNA-seq data obtained from public databases [26]. We assessed the association between TEAD family members and the prognosis of HCC patients.

### cBioPortal

cBioPortal provides a web-based database for cancer related data analysis [27]. We employed the web for understanding the biology functions in HCC tissues.

### Protein-protein interaction (PPI)

The STRING database was planned for conducting the PPInetworks [28]. Here, we established the PPI network of TEAD family using STRING and Cytoscape.

### WebGestalt

WebGestalt is an online website concentrating on enrichment analysis and supporting multiple enrichment analysis algorithms [29]. We obtained the Gene ontology (GO) enrichment analysis and Kyoto Encyclopedia of Genes and Genomes (KEGG) pathway through the web tool [30].

### TIMER2.0

The database is mainly performed to analyzing the relationship between immune infiltration and gene expression [31].

### Statistical analyses

All the results were independently repeated at least three times and represent as mean ± SD. Student’s *t*-test was employed to for proper analysis. *P* < 0.05 was regarded to be statistical significance.

## RESULTS

### TEAD family was abnormal expressed in HCC

**Figure 1:**
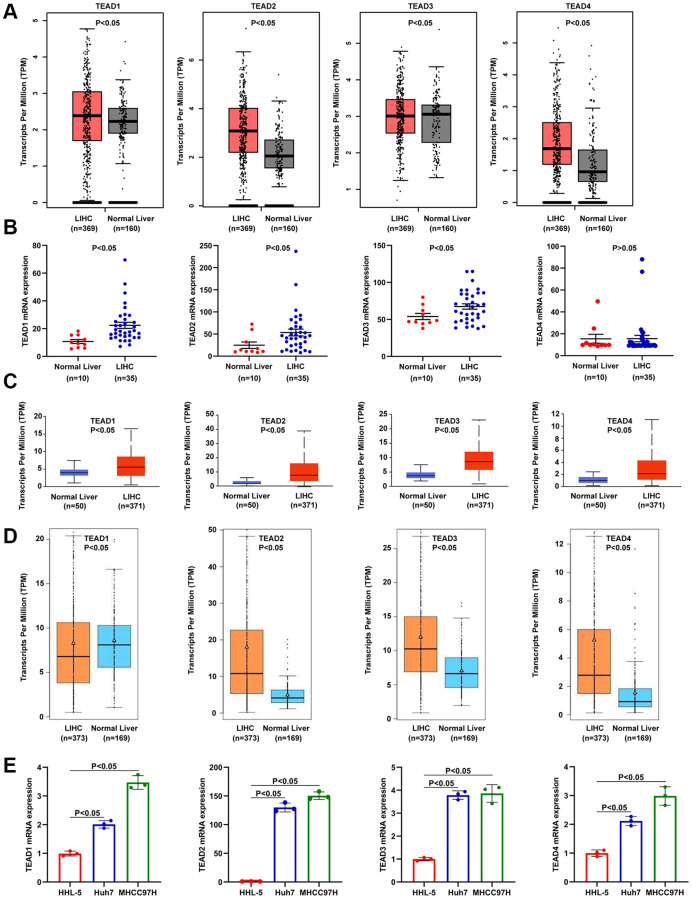
**The mRNA level of TEAD family in HCC.** (**A**–**D**) The mRNA level of TEAD members in HCC acquired from GEPIA, Oncomine, UNCLAN, and GE-mini databases. The HCC tissues and normal tissues were showed by T and N, respectively. (**E**) Levels of TEAD family members expressed in normal liver cell line HHL-5 and HCC cell lines Huh7 and MHCC97H experimented by RT-PCR.

Above all, we used the GEPIA database to explore the mRNA expression of TEAD family in HCC. TEAD1/2/4 were upregulated in HCC, while TEAD3 was downregulated (Figure 1A). In the Wurmbach Liver derived from Oncomine database, TEAD1/2/3/4 expression was increased in HCC (Figure 1B). Then, we also evaluated the expression of TEAD family in UNCLAN database, revealing that TEAD1/2/3/4 were all obviously higher in HCC to normal liver (Figure 1C). In addition, TEAD2/3/4 were evidently upregulated in HCC tissues in contrast to normal liver, however, TEAD1 was downregulated (Figure 1D). Finally, we conducted the *in vitro* experiment and RT-PCR presented that the mRNA expression of TEAD1/2/3/4 were all upregulated in Huh7 and MHCC97H cells (Figure 1E). These data illustrated that TEAD family might have roles on HCC patients.

### The prognostic of the TEAD family in HCC patients

**Figure 2:**
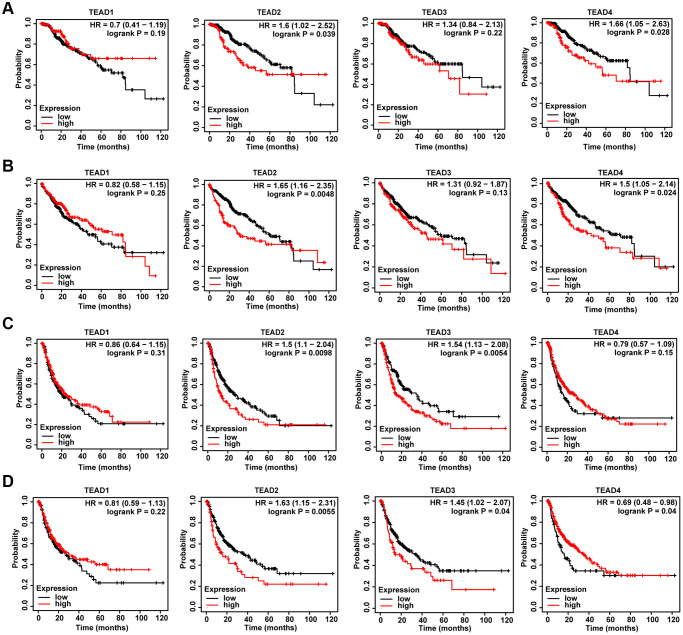
**The effect of TEAD family on the survival of HCC patients.** (**A**–**D**) The association of TEAD family with DSS, OS, PFS and RFS in HCC patients obtained from Kaplan-Meier plotter database.

Next, we discussed the influence of TEAD family on HCC patients’ survival. Based on the database, the mechanism of TEAD family expression in HCC patients’ prognosis was uncovered. As for disease-specific survival (DSS) and overall survival (OS), TEAD2/4 higher expression marked with a shorter DSS time, as well as OS time (Figure 2A, 2B). In terms of progression-free survival (PFS), the upregulated of TEAD2/3 had a powerful association with poor PFS (Figure 2C). Moreover, patients with higher expression of TEAD2/3 presented short relapse-free survival (RFS), while the lower expression of TEAD4 was related to poor RFS (Figure 2D). These results told us that TEAD family, especially TEAD2, had the potential to forecast the prognosis of HCC patients.

### Functional analysis of TEAD family associated coexpressed genes

**Figure 3:**
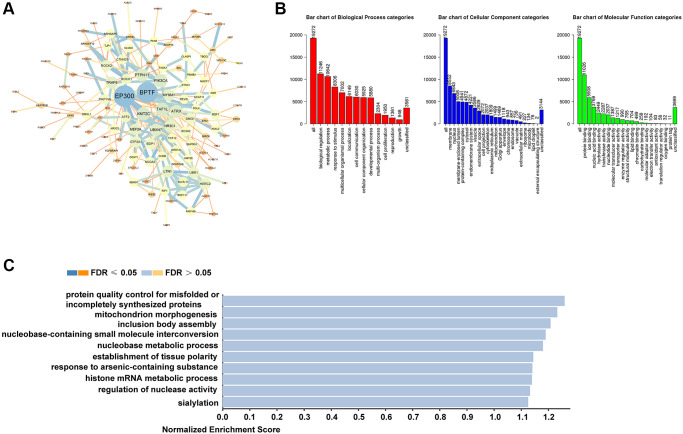
**The biological functions of TEAD family in HCC samples.** (**A**) The PPI network based on TEAD family-associated coexpression genes drew by STRING and Cytoscape. (**B**–**C**) GO and KEGG analyzed by WebGestalt.

In order to survey the exact roles of TEAD family in HCC patients, we created a comprehensive biological function analysis. From the cBioPortal database, 19272 genes were downloaded. Then, we chose the top 200 coexpressed molecules most related to TEAD family to create a PPI network by using STRING and Cytoscape software (Supplementary Table 2). Results revealed that EP300 and BPTF were mainly correlated with the TEAD family regulation (Figure 3A). Next, we employed the WebGestalt web tool to completed the GO and KEGG analysis. In terms of biological processes, the metabolism was mainly for TEAD family participated (Figure 3B). In addition, TEAD family was primarily enriched in membrane (Figure 3B). Finally, TEAD family was obviously related to nucleobase-containing small molecule interconversion and nucleobase metabolic process (Figure 3C).

### TEAD family influence on the immune cell infiltration in HCC

**Figure 4:**
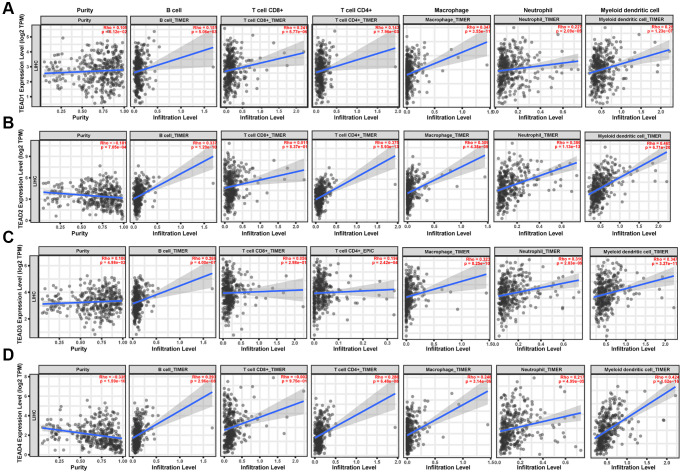
**The relationship between the expression of TEAD family and immune cell infiltration in HCC patients.** (**A**–**D**) The influence of TEAD1/2/3/4 on the immune cell infiltration.

To explore the connection between the two aspects, we acquired the information from TIMER database. Results showed that TEAD1 was positively associated with B cells, CD8+ T cells, CD4+ T cells, macrophages, neutrophils and dendritic cells (Figure 4A). Moreover, the expression of TEAD2/3/4 was positively related to the immune cells (Figure 4B–4D). Meanwhile, we further found that there was an obviously association between the clinical outcome and immune cells in HCC patients. (Table 2).

**Table 2:** The cox proportional hazard model of the *TEAD* family and six tumor-infiltrating immune cells in HCC.

	**coef**	**HR**	**95% CI_l**	**95% CI_u**	***p*.value**	**sig**
B_cell	–8.977	0	0	0.161	0.014	^*^
CD8_Tcell	–6.555	0.001	0	0.16	0.006	^**^
CD4_Tcell	–5.332	0.005	0	3.166	0.107	
Macrophage	7.24	1393.616	12.308	157797.7	0.003	^**^
Neutrophil	4.067	58.397	0.001	4825308	0.481	
Dendritic	6.745	850.031	24.212	29842.46	0	^***^
TEAD1	–0.219	0.803	0.64	1.008	0.059	
TEAD2	0.125	1.134	0.954	1.347	0.154	
TEAD3	–0.033	0.967	0.725	1.291	0.822	
TEAD4	0.11	1.117	0.91	1.37	0.29	

### TEAD2 acts as a repressor of ferroptosis

**Figure 5:**
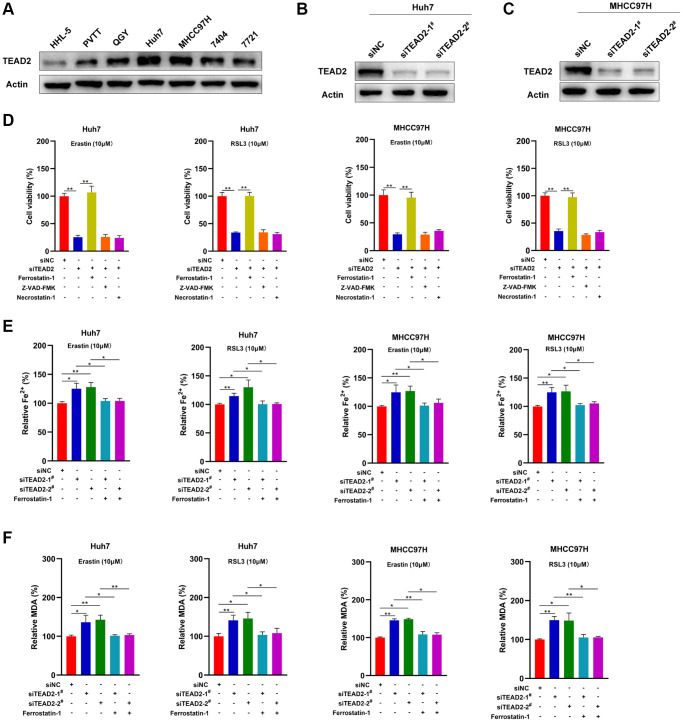
**TEAD2 negatively regulates ferroptosis in HCC.** (**A**) The protein levels of TEAD2 in normal liver cell line HHL-5 and HCC cell lines PVTT, QGY, Huh7, MHCC97H, 7404 and 7721. (**B**–**C**) Knockout of TEAD2 in Huh7 and MHCC97H cells was confirmed by Western blot. (**D**) Knocked down of TEAD2 (siTEAD2-1) promoted the cell death induced by erastin and RSL3 in Huh7 and MHCC97H cells. The cells were treated with erastin (10 μM) or RSL3 (10 μM) with or without ferrostatin-1 (5 μM), ZVAD-FMK (10 μM) and necrostatin-1 (5 μM) for 24 h. They were tested by a CCK-8 kit. (**E**–**F**) The expression levels of TEAD2 impacted on the Fe^2+^ and MDA accumulation in erastin or RSL3-treated Huh7 and MHCC97H cells. The cells were treated with erastin (10 μM) or RSL3 (10 μM) for 24 h. Subsequently, the intracellular Fe^2+^ and MDA were assayed. Data was represented with mean ± SD (*n* = 3). ^*^p < 0.05; ^**^p < 0.01.

From the above studies, we concluded that TEAD2 was upregulated in HCC. Then, we explored the protein level of TEAD2and TEAD2 was higher in PVTT, QGY, Huh7, MHCC97H, 7404 and 7721 cells, compared to HHL-5 cell (Figure 5A). Studies had shown that ferroptosis played an essential role in the biology of HCC and ferroptosis-related genes were contributed to predicting the prognosis in HCC [32]. In addition, suppressing YAP could promote HCC cells sensitive to ferroptosis [33]. Thus, we want to discuss the roles of TEAD2 in ferroptosis. Then, we knocked down TEAD2 in Huh7 and MHCC97H (Figure 5B, 5C). Next, we used the CCK-8 cell viability assay for cell death analysis after erastin and RSL3, inducers of ferroptosis, treatment in different groups. Results shown that knocked down TEAD2 could obviously promote the death of Huh7 and MHCC97H, whileferrostatin-1 could reverse the phenomenon (Figure 5D). However, ZVAD-FMK and necrostatin-1 could not effect on cell death induced by erastin and RSL3. This demonstrated that TEAD2 could inhibit the cell death through suppressing the ferroptosis in HCC cells.

**Figure 6:**
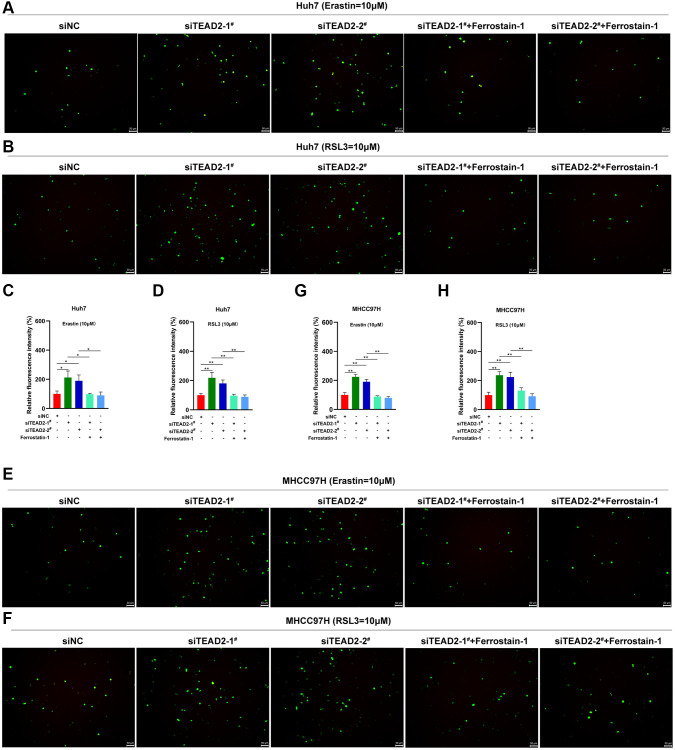
**TEAD2 regulates the ROS levels of HCC.** (**A**–**H**) The expression levels of TEAD2 impacted on the intracellular ROS levels following the treatment of erastin (10 μM) or RSL3 (10 μM) on Huh7 and MHCC97H cells. The cells were treated with erastin (10 μM) or RSL3 (10 μM) for 24 h. Subsequently, the intracellular ROS were assayed. Data was shown with mean ± SD (*n* = 3). ^**^p < 0.01.

As we all know, Fe^2+^ and lipid peroxidation were participated in ferroptosis [34]. In Figure 5E, inhibited the expression of TEAD2 increased the intracellular Fe^2+^ accumulation in both Huh7 and MHCC97H cells after erastin and RSL3 treatment, this phenomenon could be reversed by ferrostatin-1. As MDA and ROS were the crucial products of lipid peroxidation, we explored whether the indicator could be impacted by TEAD2. Results displayed TEAD2-deficient elevated the MDA levels in Huh7 and MHCC97H cells and the process could be reversed by ferrostatin-1 (Figure 5F, Figure 6A–6H). These results showed that TEAD2 overexpression could regulate Fe^2+^ accumulation and lipid peroxidation in HCC cells.

## DISCUSSION

In the study, we revealed the level and prognosis of TEAD family in HCC through bioinformatics analysis as well as experimental verification. TEAD family was abnormal expressed in HCC. In addition, the higher expression of TEAD2/4 was always had shorter DSS and OS. Patients with evaluated TEAD2/3 levels had shorter PFS and RFS, while patients with decreased TEAD4 level was connected to poor RFS. TEAD family could influence on the survival of HCC patients. Interestingly, TEAD family could influence the immune cells.

Ferroptosis is different from apoptosis and necrosis [35]. Recently, reports have shown that ferroptosis has a strongly relationship with cancer, including HCC [33]. A series of studies have demonstrated that inducing ferroptosis could promote HCC cells death and inhibit the proliferation of HCC [36]. Moreover, we also find that knock out TEAD2 could promote ferroptosis and accelerate HCC cells death. However, ferroptosis enable to promote the HCC incidence by altering tumor microenvironment [37]. These studies suggested that ferroptosis had a dual role in HCC. Nonetheless, the specific mechanisms of ferroptosis in HCC had not been clarified, which needed further research. In addition, we found that TEAD family was influenced on the immune cells. Reports have demonstrated that TEAD could coordinate several signal pathways such as Hippo, EGFR, Wnt and TGFβ [38]. The abnormal expression of TEAD could modulate several cancer-associated genes, including MYC, KRAS, NF2, BRAF and LKB1, which had crucial roles in the regulation of tumor immunity [39]. These results showed that TEAD might affect the immunological response in tumorigenesis, which deserved to be further study.

The Hippo pathway could integrate a lot of signals to directly or indirectly regulate multiple cancer hallmarks, including proliferation, invasion and metastasis, survival, angiogenesis, evading growth suppressors, as well as inflammation and immunosuppression [40, 41]. For example, the Hippo pathway could regulate the proliferation through PTEN/AKT/mTOR-mediated autophagy in HCC [42]. At present, some studies suggested that the Hippo pathway might have relationship with ferroptosis in cancer [43]. For example, Hippo-YAP/TAZ pathway could increase the sensitivity to erastin-induced ferroptosis in RCC [20]. YAP might control ferroptosis through elevated YAP protein expression in cancer [44]. Moreover, endogenous glutamate enable to enhance ferroptosis sensitivity based on the ADCY10-dependent YAP suppression in HCC [45]. In addition, YAP always binds to TEAD to promote a series of gene transcription profiles, which are involved in cell proliferation and differentiation [46]. In the study, we concluded that TEAD family could affect the development of HCC through regulating ferroptosis. These findings illustrated that the Hippo pathway could control the progression of cancer by regulating ferroptosis.

In summary, through the bioinformatics and experimental methods, we revealed the TEAD family functions on HCC. We found that the expression of TEAD2 was evidently related to OS, DSS, PFS and RFS in HCC patients. Excitedly, TEAD2 could influence the occurrence of ferroptosis. Furthermore, TEAD2 expression enabled affect immune infiltration in HCC. These results in the study showed novel biomarker for therapy and survival of HCC patients.

## Supplementary Materials

Supplementary Table 1

Supplementary Table 2
