# Supplementary material for: Integrative bioinformatics and experimental analysis revealed TEAD as novel prognostic target for hepatocellular carcinoma and its roles in ferroptosis regulation
Source: Aging (Albany NY). 2022 Jan 25;14(2):961–74. doi: 10.18632/aging.203853 (PMC8833120; doi:10.18632/aging.203853)
Supplement: Supplementary Table 1 [file aging-14-203853-s001.pdf]

SUPPLEMENTARY TABLE

Supplementary Table 1. The databases used in the analysis of the roles of *TEAD* family.

Databases	URL	Refs
GEPIA	http://gepia.cancer-pku.cn/	[22]
UALCAN	http://ualcan.path.uab.edu/analysis.html	[23]
GE-mini	http://gemini.cancer-pku.cn/	[24]
Oncomine3.0	https://www.oncomine.org/resource/login.html	[25]
Kaplan-Meier plotter	http://kmplot.com/analysis/	[26]
cBioPortal	http://www.cbioportal.org/	[27]
STRING	https://string-db.org/cgi/input.pl	[28]
WebGestalt	http://www.webgestalt.org/	[29]
KEGG	https://www.genome.jp/kegg/	[30]
TIMER2.0	https://cistrome.shinyapps.io/timer/	[31]
