# Supplementary material for: Integrative bioinformatics and experimental analysis revealed TEAD as novel prognostic target for hepatocellular carcinoma and its roles in ferroptosis regulation
Source: Aging (Albany NY). 2022 Jan 25;14(2):961–74. doi: 10.18632/aging.203853 (PMC8833120; doi:10.18632/aging.203853)
Supplement: Supplementary Table 2 [file aging-14-203853-s002.docx]

**Supplementary Table 2. The coexpression genes of *TEAD* family-associated in HCC patients.**

| **Correlated Gene** | **Cytoband** | **Spearman's Correlation** | **p-Value** | **q-Value** |
| --- | --- | --- | --- | --- |
| HIPK3 | 11p13 | 0.734436 | 3.32E-60 | 6.61E-56 |
| TTBK2 | 15q15.2 | 0.730612 | 2.70E-59 | 2.69E-55 |
| MAP3K2 | 2q14.3 | 0.720006 | 7.53E-57 | 4.56E-53 |
| STRN | 2p22.2 | 0.719629 | 9.16E-57 | 4.56E-53 |
| CCNT1 | 12q13.11-q13.12 | 0.710576 | 9.08E-55 | 3.62E-51 |
| DPP8 | 15q22.31 | 0.701829 | 6.54E-53 | 2.17E-49 |
| RAPGEF6 | 5q31.1 | 0.701504 | 7.64E-53 | 2.18E-49 |
| ZNF791 | 19p13.13 | 0.699884 | 1.66E-52 | 4.13E-49 |
| MINDY2 | 15q21.3-q22.1 | 0.695522 | 1.30E-51 | 2.87E-48 |
| PIK3CA | 3q26.32 | 0.694293 | 2.30E-51 | 4.59E-48 |
| EXOC6B | 2p13.2 | 0.693893 | 2.77E-51 | 5.02E-48 |
| ROCK1 | 18q11.1 | 0.692872 | 4.45E-51 | 7.39E-48 |
| TAOK1 | 17q11.2 | 0.689354 | 2.24E-50 | 3.43E-47 |
| RIF1 | 2q23.3 | 0.688098 | 3.96E-50 | 5.64E-47 |
| KLHL11 | 17q21.2 | 0.687164 | 6.05E-50 | 8.04E-47 |
| CLOCK | 4q12 | 0.685838 | 1.10E-49 | 1.37E-46 |
| IL6ST | 5q11.2 | 0.684339 | 2.15E-49 | 2.52E-46 |
| TRIM44 | 11p13 | 0.683903 | 2.61E-49 | 2.90E-46 |
| MYO9A | 15q23 | 0.683549 | 3.06E-49 | 3.21E-46 |
| REST | 4q12 | 0.68228 | 5.38E-49 | 5.36E-46 |
| RNF168 | 3q29 | 0.681615 | 7.22E-49 | 6.77E-46 |
| LEPROT | 1p31.3 | 0.681539 | 7.47E-49 | 6.77E-46 |
| KIAA0754 | 1p34.2 | 0.680634 | 1.11E-48 | 9.65E-46 |
| REV3L | 6q21 | 0.677081 | 5.26E-48 | 4.37E-45 |
| UHMK1 | 1q23.3 | 0.676588 | 6.51E-48 | 5.19E-45 |
| CELF1 | 11p11.2 | 0.6757 | 9.56E-48 | 7.33E-45 |
| FAM168A | 11q13.4 | 0.675262 | 1.16E-47 | 8.34E-45 |
| RASA2 | 3q23 | 0.675229 | 1.17E-47 | 8.34E-45 |
| GTF2A1 | 14q31.1 | 0.672882 | 3.21E-47 | 2.19E-44 |
| PPTC7 | 12q24.11 | 0.672816 | 3.30E-47 | 2.19E-44 |
| USP12 | 13q12.13 | 0.672375 | 3.98E-47 | 2.56E-44 |
| PIK3C2A | 11p15.1 | 0.671661 | 5.39E-47 | 3.36E-44 |
| SPATA13 | 13q12.12 | 0.671255 | 6.41E-47 | 3.87E-44 |
| ANKRD36BP1 | 1q24.2 | 0.666871 | 4.05E-46 | 2.34E-43 |
| STAG1 | 3q22.3 | 0.666774 | 4.22E-46 | 2.34E-43 |
| TCF12 | 15q21.3 | 0.666751 | 4.26E-46 | 2.34E-43 |
| LIMS1 | 2q12.3 | 0.666706 | 4.34E-46 | 2.34E-43 |
| CDKL5 | Xp22.13 | 0.666036 | 5.73E-46 | 3.01E-43 |
| CLASP1 | 2q14.2-q14.3 | 0.665789 | 6.35E-46 | 3.25E-43 |
| SECISBP2L | 15q21.1 | 0.665345 | 7.64E-46 | 3.71E-43 |
| ZFP91 | 11q12.1 | 0.665344 | 7.64E-46 | 3.71E-43 |
| NPAT | 11q22.3 | 0.664881 | 9.26E-46 | 4.39E-43 |
| RNF169 | 11q13.4 | 0.663729 | 1.49E-45 | 6.91E-43 |
| MIGA1 | 1p31.1 | 0.66359 | 1.58E-45 | 7.15E-43 |
| SBNO1 | 12q24.31 | 0.662085 | 2.93E-45 | 1.30E-42 |
| PAFAH1B2 | 11q23.3 | 0.661651 | 3.50E-45 | 1.51E-42 |
| RNF111 | 15q22.1-q22.2 | 0.661219 | 4.17E-45 | 1.77E-42 |
| ZNF641 | 12q13.11 | 0.660659 | 5.24E-45 | 2.17E-42 |
| BDP1 | 5q13.2 | 0.659373 | 8.83E-45 | 3.59E-42 |
| CREBRF | 5q35.1 | 0.659166 | 9.60E-45 | 3.83E-42 |
| JMJD1C | 10q21.3 | 0.658078 | 1.49E-44 | 5.82E-42 |
| ERCC6L2 | 9q22.32 | 0.654865 | 5.40E-44 | 2.07E-41 |
| ATRX | Xq21.1 | 0.653259 | 1.02E-43 | 3.84E-41 |
| ASXL2 | 2p23.3 | 0.652292 | 1.50E-43 | 5.44E-41 |
| KIDINS220 | 2p25.1 | 0.652285 | 1.50E-43 | 5.44E-41 |
| MAGI1 | 3p14.1 | 0.651924 | 1.73E-43 | 6.09E-41 |
| ZKSCAN8 | 6p21 | 0.651906 | 1.74E-43 | 6.09E-41 |
| DDI2 | 1p36.21 | 0.651279 | 2.23E-43 | 7.66E-41 |
| PROX1 | 1q32.3 | 0.650381 | 3.17E-43 | 1.07E-40 |
| BIRC6 | 2p22.3 | 0.649531 | 4.42E-43 | 1.47E-40 |
| UBXN7 | 3q29 | 0.648961 | 5.52E-43 | 1.80E-40 |
| MYSM1 | 1p32.1 | 0.648654 | 6.22E-43 | 2.00E-40 |
| ADAM10 | 15q21.3 | 0.648436 | 6.77E-43 | 2.14E-40 |
| ERN1 | 17q23.3 | 0.647082 | 1.14E-42 | 3.56E-40 |
| ZNF41 | Xp11.3 | 0.646463 | 1.45E-42 | 4.46E-40 |
| ETV3 | 1q23.1 | 0.645991 | 1.74E-42 | 5.27E-40 |
| TGFBRAP1 | 2q12.1-q12.2 | 0.645474 | 2.13E-42 | 6.27E-40 |
| CCDC186 | 10q25.3 | 0.64546 | 2.14E-42 | 6.27E-40 |
| NR2C2 | 3p25.1 | 0.645376 | 2.21E-42 | 6.38E-40 |
| BOD1L1 | 4p15.33 | 0.644869 | 2.68E-42 | 7.64E-40 |
| GMCL1 | 2p13.3 | 0.644742 | 2.82E-42 | 7.91E-40 |
| DPY19L3 | 19q13.11 | 0.644197 | 3.47E-42 | 9.61E-40 |
| SHPRH | 6q24.3 | 0.643982 | 3.77E-42 | 1.03E-39 |
| MGAT5 | 2q21.2-q21.3 | 0.643946 | 3.82E-42 | 1.03E-39 |
| ATF2 | 2q31.1 | 0.643867 | 3.94E-42 | 1.05E-39 |
| MAN1A2 | 1p12 | 0.642703 | 6.14E-42 | 1.61E-39 |
| HACD2 | 3q21.1 | 0.641262 | 1.06E-41 | 2.74E-39 |
| KLHDC10 | 7q32.2 | 0.641095 | 1.13E-41 | 2.89E-39 |
| ATF7IP | 12p13.1 | 0.640709 | 1.31E-41 | 3.30E-39 |
| RSF1 | 11q14.1 | 0.640392 | 1.47E-41 | 3.67E-39 |
| UEVLD | 11p15.1 | 0.640363 | 1.49E-41 | 3.67E-39 |
| ARHGAP29 | 1p22.1 | 0.638336 | 3.19E-41 | 7.75E-39 |
| DMXL1 | 5q23.1 | 0.637848 | 3.83E-41 | 9.19E-39 |
| KMT2E | 7q22.3 | 0.63768 | 4.08E-41 | 9.67E-39 |
| SEPTIN10 | 2q13 | 0.635616 | 8.79E-41 | 2.05E-38 |
| AKAP13 | 15q25.3 | 0.635596 | 8.85E-41 | 2.05E-38 |
| TTC37 | 5q15 | 0.63492 | 1.14E-40 | 2.60E-38 |
| HIVEP1 | 6p24.1 | 0.632856 | 2.43E-40 | 5.50E-38 |
| LATS1 | 6q25.1 | 0.632415 | 2.86E-40 | 6.40E-38 |
| C9ORF129 | 9q22.31 | 0.632331 | 2.95E-40 | 6.52E-38 |
| ZNF619 | 3p22.1 | 0.632268 | 3.01E-40 | 6.60E-38 |
| UBR1 | 15q15.2 | 0.631246 | 4.38E-40 | 9.49E-38 |
| NBEAL1 | 2q33.2 | 0.630275 | 6.23E-40 | 1.34E-37 |
| N4BP2 | 4p14 | 0.628857 | 1.04E-39 | 2.21E-37 |
| APOOL | Xq21.1 | 0.628222 | 1.31E-39 | 2.75E-37 |
| IPMK | 10q21.1 | 0.62813 | 1.36E-39 | 2.81E-37 |
| SCAF11 | 12q12 | 0.62749 | 1.71E-39 | 3.51E-37 |
| OSBPL8 | 12q21.2 | 0.626819 | 2.17E-39 | 4.42E-37 |
| PIKFYVE | 2q34 | 0.626123 | 2.79E-39 | 5.61E-37 |
| KIAA1109 | 4q27 | 0.625657 | 3.29E-39 | 6.56E-37 |
| PRKAR2A | 3p21.31 | 0.625426 | 3.58E-39 | 7.06E-37 |
| TOR1AIP2 | 1q25.2 | 0.625027 | 4.12E-39 | 8.06E-37 |
| MRPL14 | 6p21.1 | -0.62469 | 4.65E-39 | 9.00E-37 |
| MED13L | 12q24.21 | 0.623682 | 6.65E-39 | 1.27E-36 |
| PSMB3 | 17q12 | -0.62303 | 8.39E-39 | 1.59E-36 |
| PTAR1 | 9q21.12 | 0.622998 | 8.48E-39 | 1.59E-36 |
| KMT2C | 7q36.1 | 0.622909 | 8.75E-39 | 1.63E-36 |
| EPC1 | 10p11.22 | 0.622522 | 1.00E-38 | 1.85E-36 |
| REL | 2p16.1 | 0.62248 | 1.02E-38 | 1.86E-36 |
| HEATR5B | 2p22.2 | 0.622393 | 1.05E-38 | 1.90E-36 |
| ROCK2 | 2p25.1 | 0.621288 | 1.55E-38 | 2.78E-36 |
| PALM2AKAP2 | 9q31.3 | 0.621236 | 1.58E-38 | 2.81E-36 |
| TRAF6 | 11p12 | 0.621166 | 1.62E-38 | 2.85E-36 |
| SCYL2 | 12q23.1 | 0.621063 | 1.68E-38 | 2.93E-36 |
| DCUN1D1 | 3q26.33 | 0.621019 | 1.70E-38 | 2.95E-36 |
| RAD54L2 | 3p21.2 | 0.620874 | 1.79E-38 | 3.08E-36 |
| EMSY | 11q13.5 | 0.620363 | 2.14E-38 | 3.65E-36 |
| PTPN11 | 12q24.13 | 0.618183 | 4.59E-38 | 7.75E-36 |
| NEU3 | 11q13.4 | 0.616597 | 7.95E-38 | 1.33E-35 |
| LTN1 | 21q21.3 | 0.615625 | 1.11E-37 | 1.85E-35 |
| SON | 21q22.11 | 0.615535 | 1.15E-37 | 1.89E-35 |
| TXLNG | Xp22.2 | 0.614953 | 1.40E-37 | 2.29E-35 |
| CHD6 | 20q12 | 0.614651 | 1.56E-37 | 2.52E-35 |
| TBCEL | 11q23.3 | 0.614486 | 1.65E-37 | 2.65E-35 |
| KIF27 | 9q21.32 | 0.61401 | 1.94E-37 | 3.09E-35 |
| ARHGAP5 | 14q12 | 0.61397 | 1.96E-37 | 3.11E-35 |
| GPATCH8 | 17q21.31 | 0.613245 | 2.52E-37 | 3.95E-35 |
| LAMTOR4 | 7q22.1 | -0.61293 | 2.80E-37 | 4.37E-35 |
| SERF2 | 15q15.3 | -0.61254 | 3.20E-37 | 4.95E-35 |
| ROMO1 | 20q11.22 | -0.61215 | 3.66E-37 | 5.61E-35 |
| KRTCAP2 | 1q22 | -0.61209 | 3.73E-37 | 5.68E-35 |
| RALGAPA2 | 20p11.23 | 0.611691 | 4.27E-37 | 6.45E-35 |
| MRPL52 | 14q11.2 | -0.61132 | 4.85E-37 | 7.27E-35 |
| PKN2 | 1p22.2 | 0.611004 | 5.40E-37 | 8.03E-35 |
| ARHGEF12 | 11q23.3 | 0.610868 | 5.65E-37 | 8.35E-35 |
| NHLRC2 | 10q25.3 | 0.610702 | 5.98E-37 | 8.76E-35 |
| LMTK2 | 7q21.3 | 0.610452 | 6.51E-37 | 9.47E-35 |
| NCOA2 | 8q13.3 | 0.610233 | 7.01E-37 | 1.01E-34 |
| ZNF699 | 19p13.2 | 0.60972 | 8.33E-37 | 1.19E-34 |
| MRTFB | 16p13.12 | 0.609694 | 8.41E-37 | 1.20E-34 |
| MAN2A1 | 5q21.3 | 0.609274 | 9.68E-37 | 1.37E-34 |
| ZNF366 | 5q13.1 | 0.609122 | 1.02E-36 | 1.43E-34 |
| ATE1 | 10q26.13 | 0.609098 | 1.03E-36 | 1.43E-34 |
| CTNND1 | 11q12.1 | 0.609046 | 1.05E-36 | 1.45E-34 |
| DOCK5 | 8p21.2 | 0.608745 | 1.16E-36 | 1.59E-34 |
| MORC3 | 21q22.12 | 0.60785 | 1.56E-36 | 2.13E-34 |
| TAF1L | 9p21.1 | 0.607382 | 1.83E-36 | 2.48E-34 |
| MACF1 | 1p34.3 | 0.607161 | 1.97E-36 | 2.65E-34 |
| BPTF | 17q24.2 | 0.607136 | 1.99E-36 | 2.66E-34 |
| LMBRD2 | 5p13.2 | 0.606552 | 2.41E-36 | 3.20E-34 |
| ZNF281 | 1q32.1 | 0.605813 | 3.09E-36 | 4.07E-34 |
| GPR176 | 15q14-q15.1 | 0.605692 | 3.21E-36 | 4.21E-34 |
| RC3H2 | 9q33.2 | 0.604494 | 4.78E-36 | 6.23E-34 |
| EPC2 | 2q23.1 | 0.60404 | 5.56E-36 | 7.19E-34 |
| POLK | 5q13.3 | 0.604022 | 5.59E-36 | 7.19E-34 |
| SMG1 | 16p12.3 | 0.603023 | 7.77E-36 | 9.93E-34 |
| ARHGAP42 | 11q22.1 | 0.602837 | 8.26E-36 | 1.05E-33 |
| ERC1 | 12p13.33 | 0.601938 | 1.11E-35 | 1.40E-33 |
| CTDSPL2 | 15q15.3-q21.1 | 0.600846 | 1.59E-35 | 1.99E-33 |
| ZFR | 5p13.3 | 0.600628 | 1.71E-35 | 2.12E-33 |
| APC | 5q22.2 | 0.600499 | 1.78E-35 | 2.20E-33 |
| SLC25A40 | 7q21.12 | 0.600233 | 1.94E-35 | 2.39E-33 |
| CCDC12 | 3p21.31 | -0.6001 | 2.03E-35 | 2.48E-33 |
| RSPRY1 | 16q13 | 0.599812 | 2.23E-35 | 2.71E-33 |
| ATF7 | 12q13.13 | 0.599467 | 2.49E-35 | 3.01E-33 |
| EYA3 | 1p35.3 | 0.599428 | 2.52E-35 | 3.03E-33 |
| KLHL28 | 14q21.2 | 0.598866 | 3.03E-35 | 3.61E-33 |
| SURF2 | 9q34.2 | -0.59862 | 3.28E-35 | 3.89E-33 |
| ADAT1 | 16q23.1 | 0.598411 | 3.51E-35 | 4.14E-33 |
| HIPK1 | 1p13.2 | 0.598321 | 3.61E-35 | 4.24E-33 |
| EDF1 | 9q34.3 | -0.59812 | 3.86E-35 | 4.50E-33 |
| IREB2 | 15q25.1 | 0.59797 | 4.05E-35 | 4.69E-33 |
| MRPL55 | 1q42.13 | -0.59794 | 4.09E-35 | 4.71E-33 |
| SERINC3 | 20q13.12 | 0.597764 | 4.33E-35 | 4.96E-33 |
| EFCAB14 | 1p33 | 0.597666 | 4.47E-35 | 5.09E-33 |
| AQR | 15q14 | 0.59693 | 5.67E-35 | 6.42E-33 |
| RPS19BP1 | 22q13.1 | -0.59678 | 5.94E-35 | 6.69E-33 |
| ZNF800 | 7q31.33 | 0.596085 | 7.44E-35 | 8.33E-33 |
| NFIC | 19p13.3 | 0.595885 | 7.93E-35 | 8.83E-33 |
| TRAPPC8 | 18q12.1 | 0.595847 | 8.03E-35 | 8.89E-33 |
| MEF2A | 15q26.3 | 0.59526 | 9.69E-35 | 1.07E-32 |
| POP7 | 7q22.1 | -0.59508 | 1.03E-34 | 1.13E-32 |
| ZDHHC20 | 13q12.11 | 0.593842 | 1.53E-34 | 1.66E-32 |
| BLOC1S1 | 12q13.2 | -0.5929 | 2.06E-34 | 2.23E-32 |
| CCDC167 | 6p21.2 | -0.59225 | 2.53E-34 | 2.73E-32 |
| KIRREL1 | 1q23.1 | 0.591509 | 3.20E-34 | 3.43E-32 |
| SMG1P1 | 16p12.2 | 0.591091 | 3.65E-34 | 3.90E-32 |
| PFDN5 | 12q13.13 | -0.59079 | 4.02E-34 | 4.26E-32 |
| HERC2 | 15q13.1 | 0.5907 | 4.14E-34 | 4.36E-32 |
| SMG1P3 | 16p12.2 | 0.59026 | 4.75E-34 | 4.98E-32 |
| SLC30A4 | 15q21.1 | 0.589639 | 5.78E-34 | 6.03E-32 |
| TJP1 | 15q13.1 | 0.589533 | 5.97E-34 | 6.20E-32 |
| DHX33 | 17p13.2 | 0.588932 | 7.21E-34 | 7.45E-32 |
| ZNF524 | 19q13.42 | -0.58877 | 7.58E-34 | 7.78E-32 |
| EP300 | 22q13.2 | 0.588415 | 8.48E-34 | 8.67E-32 |
| NR3C1 | 5q31.3 | 0.588256 | 8.91E-34 | 9.06E-32 |
| SLK | 10q24.33-q25.1 | 0.5882 | 9.07E-34 | 9.18E-32 |
| ZNF689 | 16p11.2 | 0.588133 | 9.26E-34 | 9.32E-32 |
| VPS13C | 15q22.2 | 0.587486 | 1.13E-33 | 1.14E-31 |
| C17ORF49 | 17p13.1 | -0.58721 | 1.24E-33 | 1.23E-31 |
